# Home-based rehabilitation using smart wearable knee exercise device with electrical stimulation after anterior cruciate ligament reconstruction: A study protocol for a randomized controlled trial

**DOI:** 10.1097/MD.0000000000020256

**Published:** 2020-05-15

**Authors:** Gowun Kim, Won-Seok Kim, Tae Woo Kim, Yong Seuk Lee, Hooman Lee, Nam-Jong Paik

**Affiliations:** a Department of Rehabilitation Medicine, Seoul National University College of Medicine, Seoul National University Bundang Hospital, Seongnam-si; b Department of Rehabilitation Medicine, Kangwon National University Hospital, Chuncheon; c Department of Orthopedic Surgery, Seoul National University College of Medicine, SMG-SNU Boramae Medical Center, Seoul; d Department of Orthopaedic Surgery, Seoul National University College of Medicine, Seoul National University Bundang Hospital; e EXOSYSTEMS Inc., Seongnam-si, Republic of Korea.

**Keywords:** anterior cruciate ligament reconstruction, biofeedback, electrical stimulation therapy, electromyography, rehabilitation, telerehabilitation, wearable devices

## Abstract

**Introduction:**

Rehabilitation after anterior cruciate ligament (ACL) reconstruction is critical to patient outcome. Despite its importance; however, hospital-based rehabilitation is limited, with barriers, including distance and cost. With recent technological advancements, wearable devices have actively been used to address these barriers. In this study, we propose a randomized controlled trial protocol investigating the efficacy and feasibility of home-based rehabilitation after ACL reconstruction using a smart wearable device providing electrical stimulation that allows knee exercise.

**Methods and analysis:**

This is a protocol proposal for a prospective, single-center, randomized, controlled study. We plan to recruit adults discharged after ACL reconstruction; the recruited subjects will be randomly allocated to 1 of 2 groups, using a computer-generated randomization method: the intervention (n = 20) or control group (n = 20). The intervention group will receive a 6-week home-based rehabilitation program using smart wearable device. The control group will undergo a 6-week self-exercise program as normal. The following outcomes will be assessed at baseline, 2 weeks, and 6 weeks post the 6-week intervention program: quadriceps strength of the affect side as measured by a dynamometer (primary outcome); range of motion; root mean square of quadriceps muscle using surface electromyography; knee function using questionnaire; quality of life; subject's satisfaction score using questionnaire; frequency and duration of exercise; and knee pain. An intention-to-treat analysis will be conducted for the primary outcome.

**Discussion:**

This study is a prospective, single-center, randomized, controlled study. This study aims to research the feasibility and efficacy of a 6-week, structured home-based rehabilitation program for patients after ACL reconstruction using a smart wearable device. The findings of this study will help to establish a home-based rehabilitation program to better recovery in patients with ACL reconstruction.

**Trial registration number:**

This protocol was registered in ClinicalTrials.gov, under the number NCT04079205.

## 1 Introduction

Injuries and surgical interventions of the knee often lead to weakness, pain, and reduced function of the quadriceps muscle.^[1,2]^ Rehabilitation to restore the range of motion and strength of the quadriceps muscle is essential for better postoperative outcomes.^[3,4]^ However, according to a previous study, only 35% to 76% of patients comply with the exercise regimens as prescribed.^[5]^ Reasons for such a low compliance may include physical and personal problems and, more importantly, lack of patient motivation.^[6,7]^ In addition, repetitive rehabilitation exercises may be boring, and routine outpatient care may be too burdensome on patients, requiring great effort and increased cost. Moreover, lack of professional facilities and/or manpower may also contribute to the problem of low compliance. These problems collectively can ultimately result in reduced compliance and thus increase the overall rehabilitation period and reduce the effectiveness of rehabilitation treatment.^[8,9]^

To improve the overall treatment outcomes, new rehabilitation techniques that focus primarily on improving patient motivation, and thereby compliance, may be necessary. To such effort, various medical devices and rehabilitation programs have been developed, manufactured, and researched. Approaches that incorporate games or gaming techniques derived from the video game industry have been presented as inexpensive and promising alternatives to regular physical therapy or home-based rehabilitation exercises.^[10]^ In recent years, much attention has been focused on combining games and wearable technology into exercise programs, allowing patients to have more fun and an immersive experience while getting the benefits of exercise. By removing the sense of obligation by associating exercise with fun, this combination of game and wearable technology with exercise has the ability to improve patient compliance while reducing the aforementioned burden of treatment.^[11]^ Furthermore, data acquired from various sensing system in wearable devices and game scoring systems can provide more objective monitoring, which could be used to provide better feedback.^[12,13,14]^

We propose an exercise program that will incorporate wearable technology with gaming components. The wearable device that will be used in this study (exoRehab, EXOSYSTEMS Inc., Seongnam, South Korea) can measure the range of motion and strength of the knee joint. This will allow us to measure whether the exercise program can increase the range of motion and strength of the knee after the operation. Moreover, this wearable technology will also make it possible to provide biofeedback to patients to verify whether there is sufficient contract to improve knee strength by measuring the surface electromyography (EMG). Previous studies have shown that biofeedback through the surface EMG improves not only knee extension,^[15]^ but also muscle strength of the quadriceps muscle.^[16]^ In addition to providing biofeedback to patients, it is possible to provide neuromuscular electrical stimulation and rehabilitation. Surface EMG-triggered neuromuscular electrical stimulation improves knee extension via increasing the active range of motion.^[17]^ Neuromuscular electrical stimulation therapy has also been shown in previous studies to show significant improvements in muscle strength and function outcomes of the quadriceps muscles.^[18,19]^

Information from the device can be used by doctors and therapists to enhance the understanding of patient compliance and progress of knee rehabilitation, which may be useful to determine a unique, personalized treatment goal tailored to each patient. We hypothesize that the incorporation of games and wearable technology into an exercise therapy program will improve patient motivation and compliance. This approach is expected to overcome and address the shortfalls of current rehabilitation treatment methods and promote a wave of new in-home rehabilitation programs.

## 2 Methods and analysis

### 2.1 Trial design

**Figure 1:**
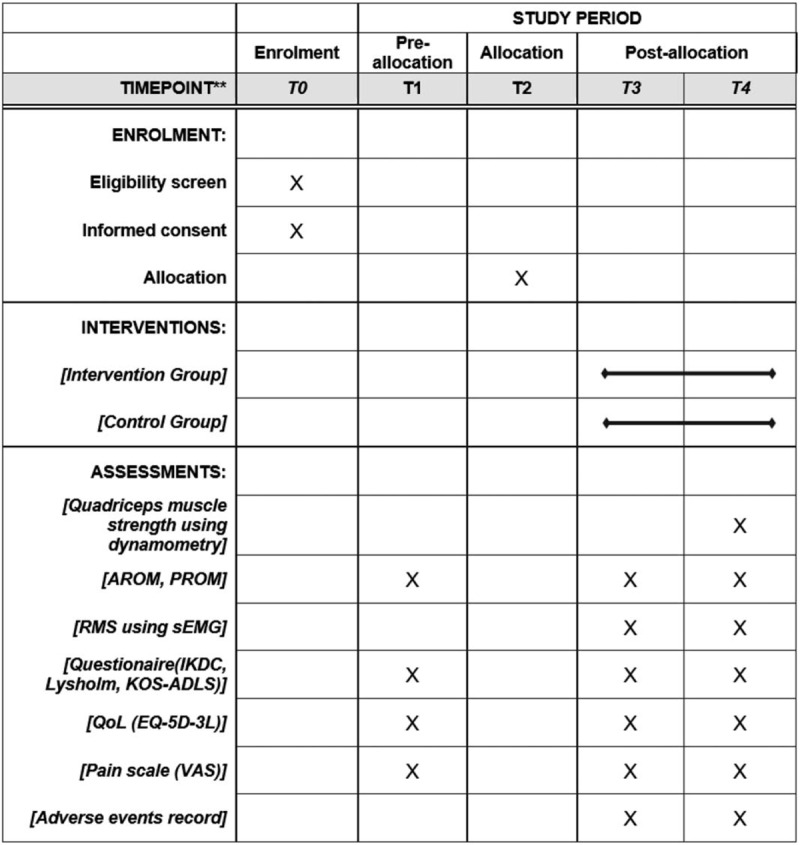
Schedule of enrolment, interventions, and assessments. T0: enrollment; T1: baseline variables; T2: allocation; T3: 2-wk after intervention initiation; T4: 6-wk after intervention initiation (endpoint of intervention, post-intervention evaluation); AROM = active range of motion, IKDC = International Knee Documentation Committee (IKDC questionnaire), KOS-ADLS = knee outcome survey activities of daily living scale, Lysholm = Lysholm knee scoring scale, PROM = passive range of motion, QoL = quality of life, RMS = root mean square, sEMG = surface electromyography, VAS = visual analog scale.

This study is a randomized controlled single-center, assessor-blinded, parallel-group clinical trial conducted at the Seoul National University Bundang Hospital, South Korea. This study was approved by the local Institutional Review Board (IRB No. B-1806-475-006) and registered in ClinicalTrial.gov (NCT04079205). The recruited participants will provide written informed consent before participation. The schedule of enrolment, interventions, and assessments is outlined in Figure 1.

### 2.2 Trial status

Data collection is currently ongoing at the time of submission study.

### 2.3 Participants

Patients who underwent anterior cruciate ligament (ACL) reconstruction at the Seoul National University Bundang Hospital will currently being screened and recruited. Subject's eligibility for inclusion will be determined during the screening process. If eligible, subjects will be assigned to 1 of the 2 groups – intervention or control group – according to the randomization method. Personal data will be numerically coded and stored in a separate database. Informed consent and all study information will be passed on to all participants. Before data collection, participants must sign the informed consent form.

#### 2.3.1 Eligibility criteria

##### 2.3.1.1 Inclusion

(1) Subjects who underwent ACL reconstruction surgery
(2) Nineteen years of age or older, with the ability to provide their own consent

##### 2.3.1.2 Exclusion (any of the following)

(1) History of surgery or traumatic injury to the uninvolved lower extremity
(2) Complication after ACL reconstruction surgery
(3) Dermatological conditions affecting the thigh
(4) Body mass index of greater than 40 kg/m^2^
(5) Implanted pacemakers or defibrillators
(6) Significant neurologic impairments
(7) Other unstable lower-extremity orthopedic conditions
(8) Initiated rehabilitation at another facility before the first physical therapy session at our facility
(9) Other lower limb impairment affecting its function
(10) More than 1 surgery for the treatment of torn ACL

### 2.4 Randomization and blinding

**Figure 2:**
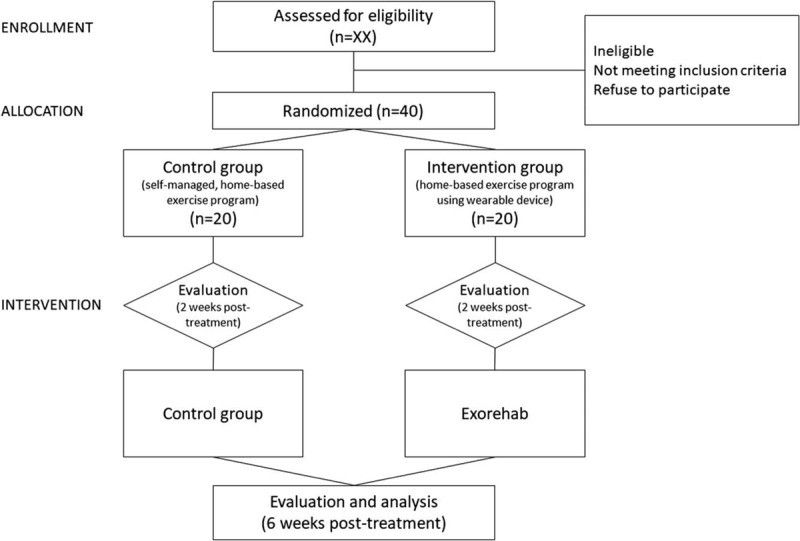
Flow diagram of the study.

Participants in this trial will be randomly assigned in a ratio of 1:1 to either the control group (home-based self-exercise program) or the intervention group (home-based exercise program using device). Participants will be allocated to 1 of the 2 groups via blocked randomization with variable size blocks to ensure an equal number of participants in both groups. This method reduces selection bias, especially in studies with small sample sizes.^[20]^ Allocation will be concealed using sequentially numbered opaque envelopes. These envelopes are stored in a lock-sealed location. A researcher without direct contact with patients will select and open the envelope in the presence of a physiotherapist to see the assignment number. This randomized assignment number will then be recorded in the case record form, and the study will be performed according to the assigned group. Due to the characteristics of this test method, the outcome assessor will also be blinded to group allocation. Once the intervention is completed, the researcher responsible for the statistical analysis will receive an Excel spreadsheet with the necessary data without any identifiable parameters. A flow diagram of the study is shown in Figure 2.

### 2.5 Intervention

After signing the informed consent forms, eligible subjects will be randomly assigned to 1 of the 2 groups: the intervention group or control group. All subjects will visit the outpatient clinic at 2 weeks, 4 weeks, and 6 weeks after the initiation of the intervention. Exercise status will be checked at these visits, and subjects will be encouraged to continue performing the exercise program.

#### 2.5.1 Device

**Figure 3:**
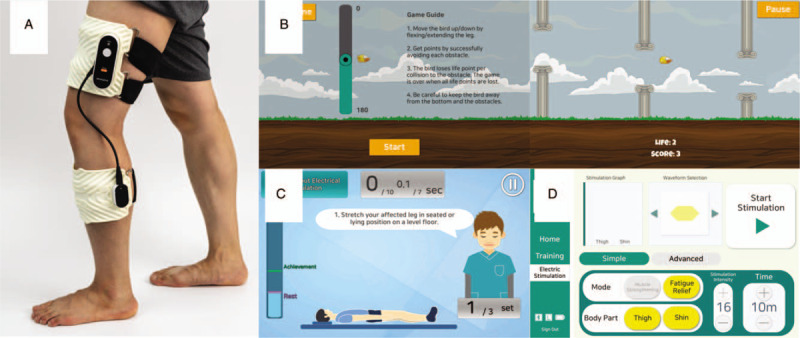
A smart wearable knee exercise and electrical stimulation device. (A) User wearing device; (B) one of the games for range of motion exercise; (C) screenshot of subject exercises using the bio-feedback – the left side of the screen shows the threshold; (D) screenshot of the neuromuscular electrical stimulation setting.

Subjects assigned to the intervention group will be provided with wearable device (Fig. 3A). The main unit of device consists of 3 parts: the thigh part, shin part, and connecting part. The program for exercise, electrical stimulation, and measurement are carried out through a separate tablet application. The tablet and main unit are connected via Bluetooth. The thigh and thin parts each have

(1) electrical stimulation electrodes for neuromuscular electrical stimulation (NMES),
(2) sensors for measuring muscle contraction through surface EMG, and
(3) sensors for measuring of knee joint angles.

In addition, data pertaining to subject's usage behavior and contents are stored on device, which can be used to analyze the progress of the knee rehabilitation program and to help set personalized treatment goals.

#### 2.5.2 Intervention group

Subjects will be educated on using the device for exercise by a physical therapist before discharge, and exercise will be performed using the device at home after discharge. Treatment may vary depending on patient's condition, but it should generally include the following:

Range of motion exercise should be repeated 3 times a day for 10 times, and it should be limited to the allowable range (Fig. 3B). The allowable range is 60° for 2 weeks, 90° for 4 weeks, and 120° for 6 weeks.

The exercises program includes quadriceps setting exercise (10 minutes, 3 times during a day), a straight leg raises exercise (5 minutes, 10 times during a day), and an ankle pump exercise (4 minutes, 10 times during a day) for 2 weeks after the operation, followed by a standing side-to-side weight shift exercise (2 minutes, 3 times during a day), a partial squat with chair exercise (2 minutes, 3 times during a day), and a heel raises exercise (2 minutes, 3 times during a day). If tolerable with this exercise program, the subject is allowed to gradually increase the frequency of the exercises. The device is designed to measure the patient's maximum voluntary isometric contraction (MVIC) before beginning a workout. To allow the patient to exercise correctly and to provide a game-like experience, the threshold will be set to 66% of MVIC, and it will be considered a count of 1 when a force above the threshold is applied in the quadriceps setting exercise program (Fig. 3C). Exercise instructions will be provided as part of pre-discharge education, and description, picture, and video of the exercises will be attached to the machine for reference. The minimum recommendation for training is about 10 minutes of quadriceps setting exercise, 3 times a day. If desired, they are allowed to practice more often.

NMES will be performed with an isometric exercise in sitting position with the knee extended. The patient will first contract the quadriceps muscles, and electrical stimulation will begin only when the force exceeds 75% of MVIC. The device delivers biphasic rectangular waveform pulses (pulse duration 400 μs, frequency of impulses 2500 Hz, train of pulses frequency 50 Hz). A total of 15 contractions is sustained for 15 seconds (inclusive of 2 seconds ramp up and down), each with 50 seconds off time. The total time for 1 set of NMES is about 22 minutes. Patients will be recommended to use NMES at least 3 times a day, about 66 minutes per session. The amplitude should be set at an intensity that is high enough to produce a full, sustained contraction of the quadriceps with visual and/or palpable evidence. The initial intensity will be set for each subject when a physical therapist trains and explains the device and exercise. The stimulus intensity will be increased further to maximum subject tolerance. As the rehabilitation program progresses, the intensity of NMES will be changed as the subject's maximal tolerable level (Fig. 3D). Exercise data using the device will automatically be stored on the device and will be used for analysis.

#### 2.5.3 Control group

Subjects in the self-exercise group (control group) will receive instructions on how to perform the exercises from a physiotherapist. The exercise program contains knee range of motion exercise, quadriceps setting exercise (10 minutes, 3 times during a day), straight leg raise exercise (5 minutes, 10 times during a day), ankle pump exercise (4 minutes, 10 times during a day), a standing side-to-side weight shift exercise (2 minutes, 3 times during a day), partial squat with chair exercise (2 minutes, 3 times during a day), and heel raise exercise (2 minutes, 3 times during a day). All exercises in the control group are the same as in the intervention group, except the use of wearable device. Consequently, biofeedback using surface EMG and electrical stimulation will not be available. Training materials will be provided, which will include an illustration of exercises with pictures. An exercise logbook will also be provided to all subjects in the self-exercise group to record exercise frequency and duration.

### 2.6 Assessment

The primary and secondary outcomes will be assessed at baseline, 2 weeks, 4 weeks, and 6 weeks postintervention (see Fig. 1). All participants will be requested to complete follow-up assessments, except for those who will withdraw or will be withdrawn from the study. Those subjects will be asked to identify reasons for withdrawal. The assessment will last approximately 1 hour, with regular breaks provided to mitigate fatigue and burden.

#### 2.6.1 Primary outcome measures

Isokinetic strength testing will be performed using the dynamometer (Primus RS, BTE Technologies, Hanover, MD) at 6 weeks after the intervention. We will report the ratio (as a percentage) of knee extensor strength in the affected side to the unaffected side.

#### 2.6.2 Secondary outcome measures

- Active and passive range of motion of the knee
- Root mean square of surface EMG (measured bilateral rectus femoris, vastus medialis, and vastus lateralis)
- Knee function or deficiency using questionnaire (International Knee Documentation Committee score,^[21]^ Lysholm score,^[22,23]^ Knee outcome survey activities of daily living^[24]^)
- Quality of life (European Quality of Life-5 Dimensions 3-Level version^[25]^)
- Pain evaluation (visual analog scale)
- Satisfaction evaluation (5-point Likert scale)
- Frequency and duration for exercise using data from device or exercise logbook

### 2.7 Harms and benefits

The medical device used in this study has the ability to measure the range of motion exercises, providing biofeedback through surface EMG measurement and neuromuscular electrical stimulation therapy. Each method is a commonly used treatment method for rehabilitation. The range of motion exercises and biofeedback through surface EMG measurement are not expected to be inconvenient or risky to the user. Neuromuscular electrical stimulation therapy is an electrical stimulation via medical electrodes. Since there are no reports that document any side effects or complications from neuromuscular electrical stimulation therapy, safety is not a major concern.^[26]^ Although there may be subjective discomfort in performing neuromuscular electrical stimulation therapy, the intensity can be controlled by the subject; instructions for controlling the intensity will be adequately explained. If there is a situation that requires termination of the therapy due to discomfort or other medical or subjective reasons, they will be withdrawn from the study immediately and reasons for discontinuation will be recorded.

### 2.8 Criteria for discontinuation

(1) Voluntary withdrawal of consent from the trial.
(2) Aggravation of underlying disease.
(3) Incompliance to intervention procedures (in this instance a specific reason must be recorded by the investigator).
(4) Participation in the study is thought to interfere with other medical procedures.

### 2.9 Data and safety monitoring plan

A data and safety monitoring plan is established. Adverse events and data on the subjects’ assessments will be monitored. Data will be accessible only by the study team. Any hardcopy documentation will be stored in locked cabinets in locked offices. Data and safety monitoring committee (DSMC) guided by a prepared charter of roles and responsibilities. The DSMC will meet every 3 months to review recruitment, collected study data, and adverse events and will guide the study team regarding any needed action. Unanticipated events, adverse effects of the study subjects, and cases of noncompliance with the research protocol are reported to the IRB within a specified period. Major noncompliance, adverse events, and unintended events will be reported within 15 days. Minor noncompliance will be reported every 3 months.

### 2.10 Statistical issues

#### 2.10.1 Sample size calculation

This study was designed as an exploratory trial with 2 groups – intervention and control groups – using a dynamometer. The sample size calculation was based on the difference in the quadriceps strength (torque, Nm) between the 2 groups from the baseline to the 6-week follow-up. The following formula was used to calculate the number of participants needed in each group:
 
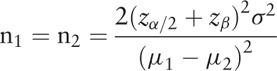


According to a previous study, the difference in the means of 6-week quadriceps strength was 33, with a standard deviation of 30.^[27]^ Based on the 5% 2-sided significance level and 80% power, at least 14 participants are needed in each group. With an attrition rate of 30%, our study will recruit a total of 20 participants in each group.

### 2.11 Statistical analysis

The analysis will be described for all results included in the study. Quantitative outcomes will be expressed with their mean ± standard deviation, and qualitative results will be described with their absolute value, percentage, and 95% confidence intervals.

Data will be analyzed using SPSS V.22.0 software. To observe the associations between the qualitative variables, *χ*2 test will be used. Before comparing the means, Kolmogorov–Smirnov test and the Levene test will be used to confirm the normal distribution and homogeneity of the variances of the quantitative variables. For variables with normal distribution, Student t-test will be used; for variables with a non-normal distribution, Mann–Whitney test will be used. Analysis of variance mixed model will be used to estimate of the average effect (differences between groups) for all variables. This analysis model incorporated the groups (intervention and control group), time (baseline, 2 weeks and 6 weeks), and the group × time interaction. When a significant *F* value is found, the Bonferroni post-hoc test will be applied to identify the differences. The size of the effect between the groups for the variables that present intergroup differences can be calculated at some point, with 95% confidence intervals. The level of statistical significance adopted will be 5%. The analysis will be performed by an independent researcher.

### 2.12 Patient and public involvement

Patients or the public were not involved in the design of this study. Informed consent and all study information will be given to all participants. Only after willingly consenting and understanding of the study, patients will be randomized. The results of the assessment can be released to participants on request.

### 2.13 Ethics and dissemination

This protocol has been approved by the IRB of the Seoul National University Bundang Hospital (IRB No. B-1806-475-006). The trial will be conducted in accordance with the International Conference on Harmonisation Good Clinical Practice. All study procedures will adhere to the Declaration of Helsinki. The primary results will be submitted for publication to an international, peer-reviewed journal, regardless of whether the results are positive, negative, or inconclusive with respect to the study hypothesis. Authorship eligibility will be based on the recommendations from the International Committee of Medical Journal Editors. Any important protocol amendments will be reported to the IRB of the Seoul National University Bundang Hospital and registered at ClinicalTrials.gov.

## 3 Discussion

This study aims to investigate the feasibility and efficacy of a 6-week, structured home-based rehabilitation program for patients after ACL reconstruction using smart wearable device, which is an all-in-one home-based rehabilitation platform (range of motion exercise, strengthening using EMG biofeedback, electrical stimulation). This study is a prospective, single-center, randomized, controlled study. Subjects will be randomly allocated to 1 of 2 groups, the intervention (n = 20) or control group (n = 20). The following outcomes will be assessed at baseline, 2 weeks, and 6 weeks post the 6-week intervention program: quadriceps strength of the affect side as measured by a dynamometer (primary outcome); range of motion; root mean square of quadriceps muscle using surface EMG; knee function and subject's satisfaction score using questionnaire; quality of life; frequency and duration of exercise; and knee pain. It is hypothesized that patients receiving this rehabilitation program with device will show superior improvement in quadriceps muscle strength.

## Author contributions

GK, W-SK, and TWK led the study design, and designed and planned the statistical analysis. GK conceived the study, developed the protocol, setting, and wrote the first draft of the manuscript. N-JP and W-SK conceived the study, wrote the grant, developed the protocol, setting, and reviewed the manuscript. HL was the main creator of the device carrying out the electronic design, which includes hardware and software. YSL and TWK contributed in enrollment of participants and data collection. GK, W-SK, TWK, YSL, HL, and N-JP reviewed the manuscript critically and gave the final approval for publication.
